# Curcumin reversed chronic tobacco smoke exposure induced urocystic EMT and acquisition of cancer stem cells properties via Wnt/*β*-catenin

**DOI:** 10.1038/cddis.2017.452

**Published:** 2017-10-05

**Authors:** Zhaofeng Liang, Ling Lu, Jiahui Mao, Xia Li, Hui Qian, Wenrong Xu

**Affiliations:** 1 Key Laboratory of Medical Science and Laboratory Medicine of Jiangsu Province, School of Medicine, Jiangsu University, Zhenjiang, China; 2 Department of Children's Health Care, Women and Children Health Hospital of Zhenjiang, Jiangsu Province, Zhenjiang, China

## Abstract

Tobacco smoke (TS) is the most important single risk factor for bladder cancer. Epithelial–mesenchymal transition (EMT) is a transdifferentiation process, involved in the initiation of TS-related cancer. Cancer stem cells (CSCs) have an essential role in the progression of many tumors including TS-related cancer. However, the molecular mechanisms of TS exposure induced urocystic EMT and acquisition of CSCs properties remains undefined. Wnt/*β*-catenin pathway is critical for EMT and the maintenance of CSCs. The aim of our present study was to investigate the role of Wnt/*β*-catenin pathway in chronic TS exposure induced urocystic EMT, stemness acquisition and the preventive effect of curcumin. Long time TS exposure induced EMT changes and the levels of CSCs’ markers were significant upregulated. Furthermore, we demonstrated that Wnt/*β*-catenin pathway modulated TS-triggered EMT and stemness, as evidenced by the findings that TS elevated Wnt/*β*-catenin activation, and that TS-mediated EMT and stemness were attenuated by Wnt/*β*-catenin inhibition. Treatment of curcumin reversed TS-elicited activation of Wnt/*β*-catenin, EMT and CSCs properties. Collectively, these data indicated the regulatory role of Wnt/*β*-catenin in TS-triggered urocystic EMT, acquisition of CSCs properties and the chemopreventive effect of curcumin.

Bladder cancer is the ninth most common malignancy, with an estimated 429 793 new diagnosed cases and 165 084 deaths every year worldwide.^1, 2^ In China, bladder cancer is the first leading causes of cancer-related death among urinary malignancies.^3^ Although the causes of bladder cancer are not well known, bladder cancer has been linked to tobacco smoke (TS), parasitic infection, exposure to radiation or chemicals and other risk factors. Studies have shown that TS is strongly associated with the occurrence and development of bladder cancer.^3, 4^ It is reported that TS is the most important single risk factor for bladder cancer with 40–60% causally related.^4^ Smokers have an estimated fourfold higher risk of bladder cancer than nonsmokers.^5^ Accumulating evidence suggested that epithelial–mesenchymal transition (EMT) and the acquisition of cancer stem cells (CSCs) properties is an important underlying mechanisms for initiation, invasion and metastasis of cancer. However, the mechanisms leading to EMT and stemness acquisition are not fully understood, which has hindered the development of effective targeted therapies and the chemoprevention of cancers including bladder cancer.

EMT is a reversible naturally occurring transdifferentiation process consisting in the changes from an epithelial to a migratory mesenchymal cell phenotype. EMT has been shown to be associated with the progress of cancer, the metastatic spread and progression of cells from the site of the primary tumor to the surrounding tissues and distant organs.^6, 7, 8^ TS has been documented to promote EMT.^9, 10, 11^ TS-induced EMT has been found to regulate early events in cancer progression such as downregulation of epithelial cadherin, loss of cell junctions and apical–basal polarity, and enabling cells motility.^11^ However, the underlying mechanisms regarding how TS induces EMT and the signaling events involved remains poorly understood.

The recently uncovered link between passage through EMT and acquisition of CSCs properties indicates that the EMT programs serves as one of the mechanisms for generating CSCs.^12, 13, 14^ Increasing evidences have confirmed that many cancers including bladder cancer arise from a small sub-population of cancer cells, which are termed CSCs.^15, 16^ CSCs with the properties of self-renewing and multipotent differentiation have an important role in human cancers progression. CSCs are also implicated in formation of metastases and relapse of malignancies. Although our previous studies showed that exposed the SV-40 immortalized human urothelial cell line (SV-HUC-1) to TS induces EMT,^2, 17^ it has not been determined whether long-term TS exposure induces EMT and thereby contributes to the acquisition of CSCs properties and malignant transformation of bladder cells *in vitro* and *in vivo*.

Wnt/*β*-catenin signaling pathway is an evolutionarily conserved signaling pathway and critical for a variety of biological processes.^18^ Numerous studies have suggested the important functions of Wnt/*β*-catenin signaling in several cancers.^19^ In the absence of Wnt stimulation, *β*-catenin is phosphorylated by a destruction complex consisting of GSK3*β*, CK1, Axin1, Axin2 and APC, resulting in the *β*-catenin degradation. Upon binding of Wnt, phosphorylation of *β*-catenin is blocked and allows *β*-catenin to dissociate from the destruction complex. Then, *β*-catenin accumulates in cytoplasm that results in translocation of *β*-catenin to the nucleus and interacts with TCF/LEF to transactivate the downstream target genes including Cyclin D1, c-Myc, CD44 and ALDH *etc*.^19, 20^ Wnt/*β*-catenin signaling pathway is an important inducer of EMT and critical for the maintenance of CSCs. Upon activation by specific ligands, *β*-catenin is released from the membrane and promotes transcription of genes involved in mesenchymal phenotype induction and the maintenance of CSCs.^21^ However, the role of Wnt/*β*-catenin in the long time TS exposure induced urocystic EMT and acquisition of CSCs properties still has not been defined.

Recently, some studies have illustrated that dietary phytochemicals with potent anticancer activity presented in consuming food-based dietary. It has been reported that approximately one-third of cancers can be prevented by controlling diet and regular physical activities. These effects partly have been linked to their suppressing effect on the EMT, CSCs characteristics, consequently invasion and metastasis.^22^ Curcumin, a yellow polyphenolic compound, is the principal active component of the spice turmeric. It has long been used throughout Asia as a food additive and a traditional herbal medicine. Evidences obtained from *in vitro* and *in vivo* studies indicated that curcumin has a therapeutic potential in preventing and treating several types of cancer.^3, 23^ The ability of curcumin for the target CSCs and modulation associated pathways has been proposed in several studies.^24, 25^ Accumulating evidences also showed that the potent chemopreventive activity of curcumin may be partly derived from the inhibition of EMT.^26, 27^

The aim of this study was to investigate the role of Wnt/*β*-catenin pathway in chronic TS exposure induced urocystic EMT, stemness acquisition, and the preventive effect of curcumin *in vitro* and *in vivo*. Our results indicated that curcumin reverse the long-term TS exposed induced urocystic EMT, the acquisition of CSC-like properties and these effects are modulate associated with the inhibition of the Wnt/*β*-catenin pathway. These findings provide new insights into the pathogenesis and the chemoprevention of TS-associated bladder cancer.

## Results

### Long-term smoke exposure induced malignant transformation of SV-HUC-1 cells

**Figure 1:**
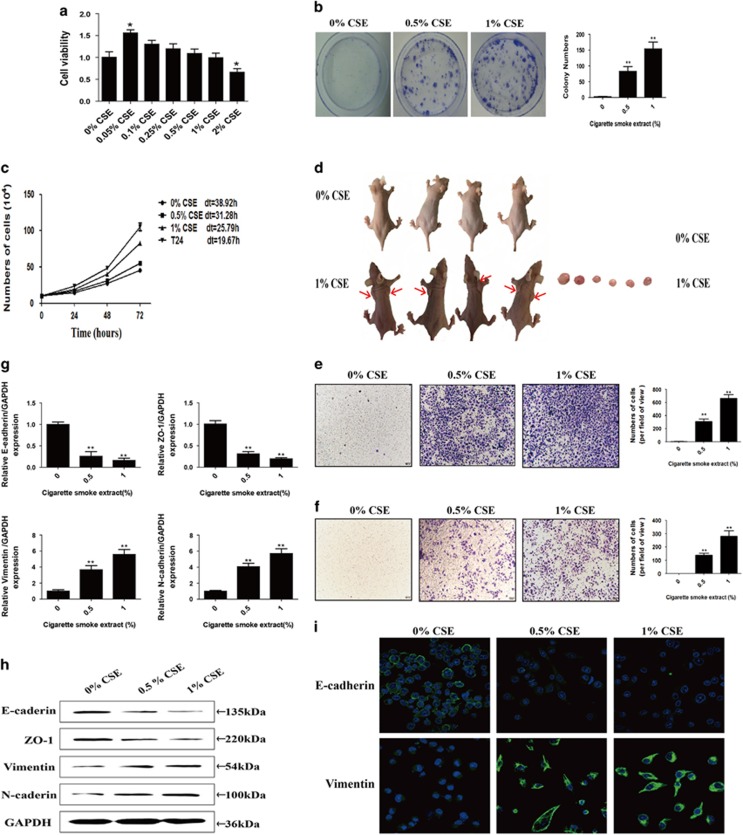
Chronic CSE exposure induced malignant transformation and EMT of SV-HUC-1 cells. (**a**) Cells viabilities were examined after exposed to various concentrations of CSE for 14 days. (**b**) Cell colonies and their numbers of SV-HUC-1 cells and CSE-transformed SV-HUC-1 cells. (**c**) Cell doubling time of SV-HUC-1 cells and CSE-transformed SV-HUC-1 cells. (**d**) CSE-transformed SV-HUC-1 cells (1 × 10^6^ cells) were injected into nude mice to observe the tumorigenicity (*n*=4). (**e** and **f**) Chronic CSE exposure enhanced migratory and invasive capacities of SV-HUC-1 cells, as determined by transwell migration and invasion assays. (**g**-**i**) CSE decreased the expression of epithelial markers, and increased the expression of mesenchymal markers. Data are expressed as mean±S.D. **P<0.01, compared with control group

TS is one of the most important risk factor for bladder cancer and can also induce malignant transformation of cells.^28^ To investigate the effects of cigarette smoke extract (CSE) on cell transformation, the SV-40 immortalized human urothelial cells (SV-HUC-1) were first exposed to various concentrations of CSE (0, 0.05, 0.1, 0.25, 0.5, 1 or 2%) for 14 days. Cells viabilities were decreased by concentrations of 2% after 14 days exposure (Figure 1a). Therefore, the cells were routinely exposed to CSE at concentrations of 0.5 or 1% for following experiments, the maximum concentrations causing no changes in cells viabilities.

SV-HUC-1 cells were chronically exposed to CSE (0, 0.5 or 1%) for about 20 weeks (48–72 h per passage). Then, we establish their doubling time and capacity for independent clone formation, the characteristics of malignant transformed cells. Colony-forming assays shown that 103±18 colonies were formed by SV-HUC-1 cells exposed to 0.5% CSE, and 182±22 colonies were formed by SV-HUC-1 cells exposed to 1% CSE. In contrast, normal SV-HUC-1 cells showed no independent clone formation (Figure 1b). We also found that chronically exposed to CSE elevated the proliferation of cells and shorten cells doubling time (Figure 1c). These were confirmed by the xenograft assays in which SV-HUC-1 cells and CSE-transformed cells were injected into nude mice. Results showed that CSE-transformed cells significantly increased the tumor incidence rate (Figure 1d).

### Chronic CSE exposure induced EMT and acquisition of CSCs properties during the cells transformation

TS-induced EMT is critically involved in TS-associated malignant transformation. To evaluate the effect of chronic CSE exposure on EMT, transwell assays were carried out to analyze the migratory and invasive capacities of SV-HUC-1 cells. The results showed that CSE treatment significantly increased SV-HUC-1 cells migration and invasive capacities (Figures 1e and f). To further examine whether molecular alterations of EMT occurred in long-term CSE-transformed cells, the expression levels of EMT markers were determined (Figures 1g and i). After chronic exposure of cells to CSE, the expression levels of the epithelial markers such as E-cadherin and ZO-1 were decreased. In contrast, expression levels of the mesenchymal markers, Vimentin and N-cadherin, were increased. Immunofluorescent staining also showed that CSE decreased E-cadherin protein expression and increased Vimentin expression in SV-HUC-1 cells.

**Figure 2:**
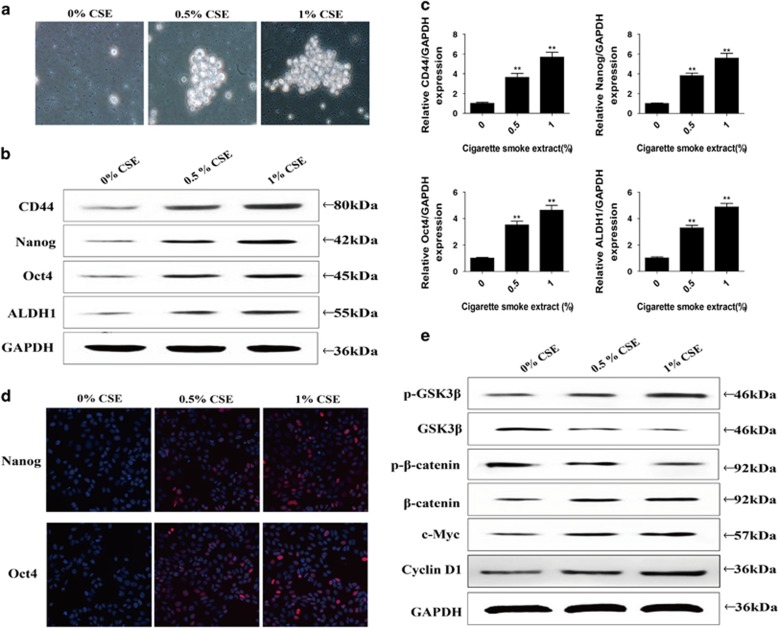
Chronic CSE exposure induced acquisition of CSCs properties and activation of Wnt/*β*-catenin in SV-HUC-1 cells. (**a**) The images of spheroids that were seeded by CSE-transformed SV-HUC-1 cells. (**b-d**) Chronic CSE exposure increased the expression of specific cell surface markers of CSCs. (**e**) CSE increased p-GSK3*β*, *β*-catenin, c-Myc and cyclin D1 expression levels and decreased the expression of GSK3*β* and p-*β*-catenin. Data are expressed as mean±S.D. **P<0.01, compared with control group

In general, the EMT program tends to cause an increased expression of genes associated with 'stemness', CSCs numbers in normal or tumor cells and initiation of tumors. Whether long-term CSE exposure has a role in CSC generation need to be determined. To address this issue, a sphere formation assay was performed to evaluate the potential for self-renewal, the mRNA and protein levels of specific cell surface markers of bladder CSCs were measured. In chronic CSE exposed SV-HUC-1 cells, there was an increase in formation of spheroids (Figure 2a). As measured by qRT-PCR and western blot analyses, during the CSE-induced EMT, levels of CSCs markers (CD44, Nanog, Oct4 and ALDH1) were significantly upregulated (Figures 2b and d).

### CSE-induced EMT and obtained CSCs properties were associated with Wnt/*β*-catenin

EMT, stem cell-like population, and the cancer growth driving is intimately related to Wnt/*β*-catenin pathway. To determine whether CSE-elicited urocystic cells EMT and the acquisition of CSC-like properties were associated with changes of Wnt/*β*-catenin pathway, the levels of p-GSK3*β*, GSK3*β*, p-*β*-catenin, *β*-catenin, c-Myc and cyclin D1 were measured. Chronic exposure of SV-HUC-1 cells to CSE increased p-GSK3*β*, *β*-catenin, c-Myc and cyclin D1 expression levels and decreased the expression of GSK3*β* and p-*β*-catenin (Figure 2e). These results suggested that Wnt/*β*-catenin pathway is involved in the acquisition of CSC-like properties of CSE-transformed SV-HUC-1 cells.

### Activation of Wnt/*β*-catenin mimics CSE-induced EMT and CSCs properties

**Figure 3:**
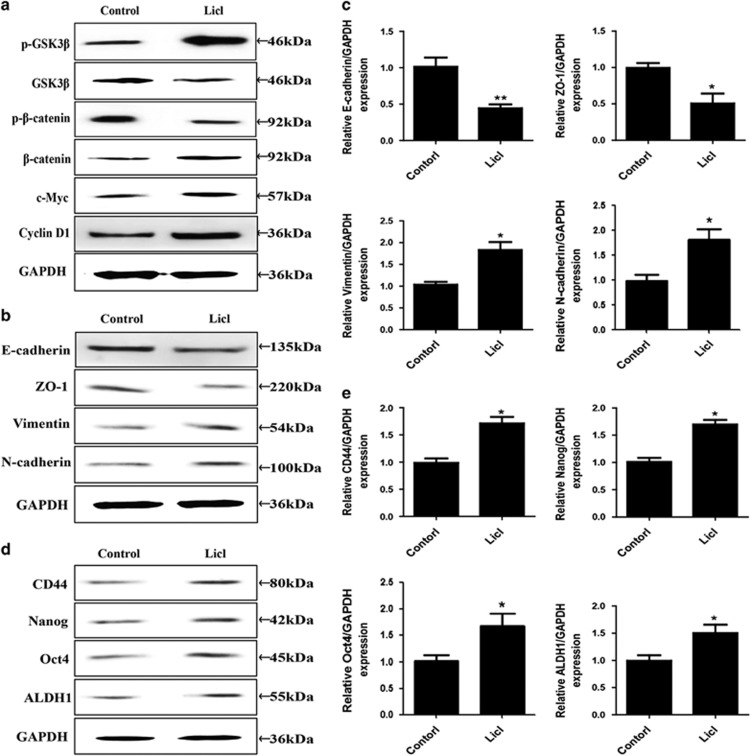
Activation of Wnt/*β*-catenin mimics CSE-induced EMT and CSCs properties in SV-HUC-1 cells. (**a**) Licl activated Wnt/*β*-catenin activation in SV-HUC-1 cells. (**b** and **c**) Licl altered the expression of EMT markers. (**d** and **e**) Licl increased the expression levels of CSCs markers. Data are expressed as mean±S.D. *P<0.05, **P<0.01

To examine the role of Wnt/*β*-catenin pathway in the long-term CSE-induced EMT and acquisition of CSC-like properties in SV-HUC-1 cells, a Wnt/*β*-catenin pathway activator (Licl) was used to activate Wnt/*β*-catenin pathway. We showed that LiCl treatment inactivated GSK3*β* and increased the expression of *β*-catenin c-Myc and cyclin D1 (Figure 3a). Our data also showed that LiCl treatment resulted in higher expression of CSCs markers (CD44, Nanog, Oct4 and ALDH1) and EMT-like changes (Figures 3b and e). Thus, these results suggested that Wnt/*β*-catenin pathway may have an important role in CSE-induced EMT and acquisition of CSCs properties.

### Wnt/*β*-catenin suppression reversed long-term CSE exposure-triggered EMT and CSCs properties

**Figure 4:**
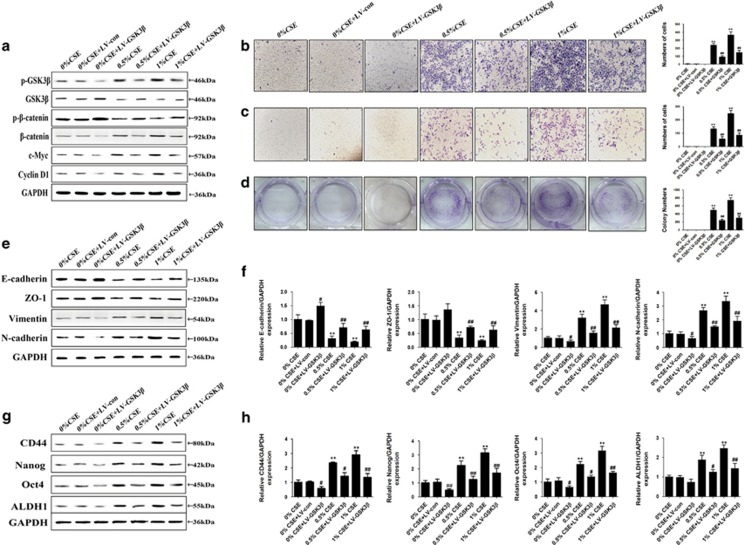
Wnt/*β*-catenin suppression attenuated chronic CSE exposure-triggered EMT and acquisition of CSCs properties. (**a**) Transfection of GSK3*β* overexpression lentiviral vector ameliorated CSE-induced activation of Wnt/*β*-catenin. (**b** and **c**) Wnt/*β*-catenin suppression reversed CSE enhanced migratory and invasive capacities. (**d**) Cell colonies and their numbers. (**e** and **f**) Wnt/*β*-catenin inhibition attenuated CSE-induced decreases of the levels of epithelial markers, and increased the expression of mesenchymal markers. (**g** and **h**) Wnt/*β*-catenin inhibition ameliorated CSE-induced increases in expression of CSCs markers. LV-con, lentiviral control vector; LV-GSK3*β*, lentiviral expression vector for GSK3*β*. **P<0.01, compared with control group; ^#^*P*<0.05, ^##^*P*<0.01, compared with CSE groups

As above results revealed that CSE-induced EMT and acquisition of CSC-like properties were associated with activation of Wnt/*β*-catenin in CSE-transformed SV-HUC-1 cells, we further explored the role of Wnt/*β*-catenin pathway in this process. SV-HUC-1 cells were transfected with GSK3*β* overexpression lentiviral vector at multiplicity of infection (MOI) of 5, 10, 15, 30 and 50 for 72 h. Green fluorescent protein (GFP) fluorescence imaging showed the successful transfection of SV-HUC-1 cells with a MOI of 30 as the optimal infection efficiency. Results showed that table transfection of GSK3*β* with lentiviral vector restored CSE-suppressed GSK3*β* activity and attenuated CSE-triggered activation of Wnt/*β*-catenin in SV-HUC-1 cells (Figure 4a). Overexpression of GSK3*β* decreased CSE-mediated migration and invasion capacities of SV-HUC-1 cells (Figures 4b and c). Overexpression of GSK3*β* reversed CSE enhanced the capacity for clone formation (Figure 4d). GSK3*β* overexpression vector attenuated CSE-induced decrease of E-cadherin and ZO-1 levels, as well as increase of Vimentin and N-cadherin in SV-HUC-1 cells (Figures 4e and f). In addition, western blot and qRT-PCR analyses showed that transfection of GSK3*β* with lentiviral vector attenuated CSE-induced the increase of CD44, Nanog, Oct4 and ALDH1 (Figures 4g and h). Together, these data demonstrated the regulation of Wnt/*β*-catenin on long-term CSE exposure-triggered EMT and CSC-like properties of SV-HUC-1 cells.

### Curcumin suppressed Wnt/*β*-catenin to reverse CSE-induced urocystic EMT and stemness

**Figure 5:**
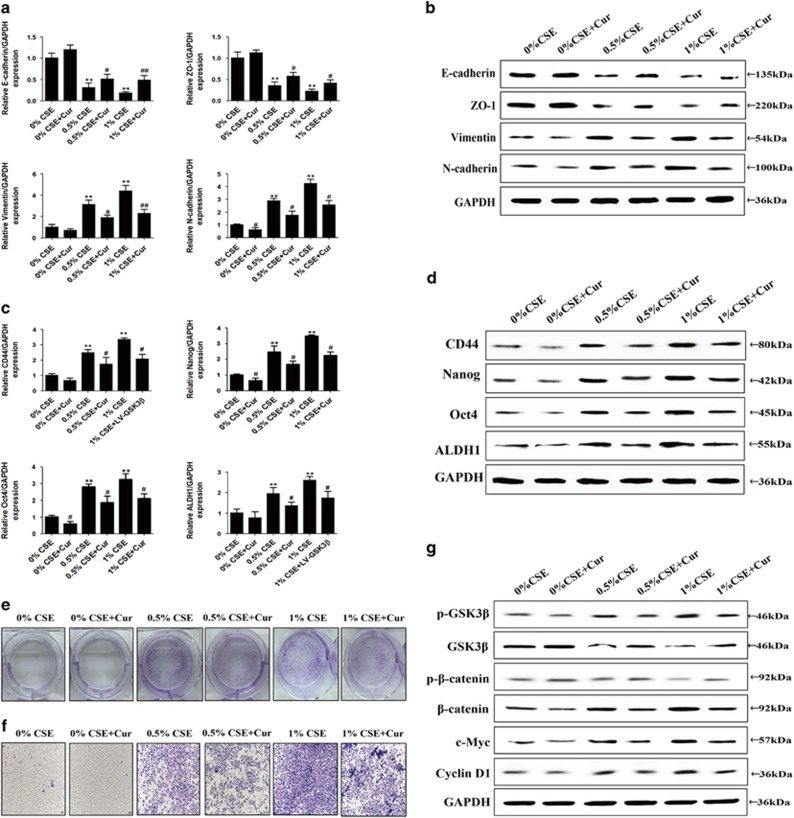
Curcumin reversed CSE-induced urocystic EMT and stemness via Wnt/*β*-catenin. (**a** and **b**) Curcumin attenuated CSE-induced decreases of the levels of epithelial markers, and increased the expression of mesenchymal markers. (**c** and **d**) Curcumin attenuated CSE-induced increases in expression levels of CSCs markers. (**e**) Curcumin reversed CSE enhanced the capacity for clone formation. (**f**) Curcumin attenuated CSE enhanced migratory and invasive capacities. (**g**) Curcumin ameliorated CSE-induced activation of Wnt/*β*-catenin. **P<0.01, compared with control group; ^#^*P*<0.05, ^##^*P*<0.01, compared with CSE groups

In order to determine the effects of curcumin on CSE-mediated urocystic EMT and CSC-like properties, CSE-transformed SV-HUC-1 cells were treated with curcumin (10 *μ*M) for 7 days. Our results showed that CSE-induced alterations in mRNA and protein expression levels of the EMT markers, including decrease of the epithelial markers (E-cadherin and ZO-1), and increase of the mesenchymal markers (Vimentin and N-cadherin), were significantly attenuated with curcumin (10 *μ*M) treatment (Figures 5a and b). Curcumin reversed CSE-mediated migration and clone formation capacities of transformed SV-HUC-1 cells (Figures 5e and f). Figures 5c and d show that CSE-induced increase of CD44, Nanog, Oct4 and ALDH1 were effectively suppressed by curcumin. These data indicated that curcumin reversed CSE-induced urocystic EMT changes and CSC-like properties in CSE-transformed SV-HUC-1 cells. To explore the influence of curcumin on CSE-mediated urocystic activation of Wnt/*β*-catenin pathway, we further examined the changes in Wnt/*β*-catenin activation following curcumin treatment. Western blot analyses showed that curcumin inhibited long terms CSE-induced alterations in expression levels of Wnt/*β*-catenin pathway, including decrease of GSK3*β*, p-*β*-catenin and increases of p-GSK3*β*, *β*-catenin, c-Myc and cyclin D1 were significantly reversed by curcumin treatment. (Figure 5g).

### Long-term TS exposure activated Wnt/*β*-catenin and altered the expression of EMT and CSC markers *in vivo*

**Figure 6:**
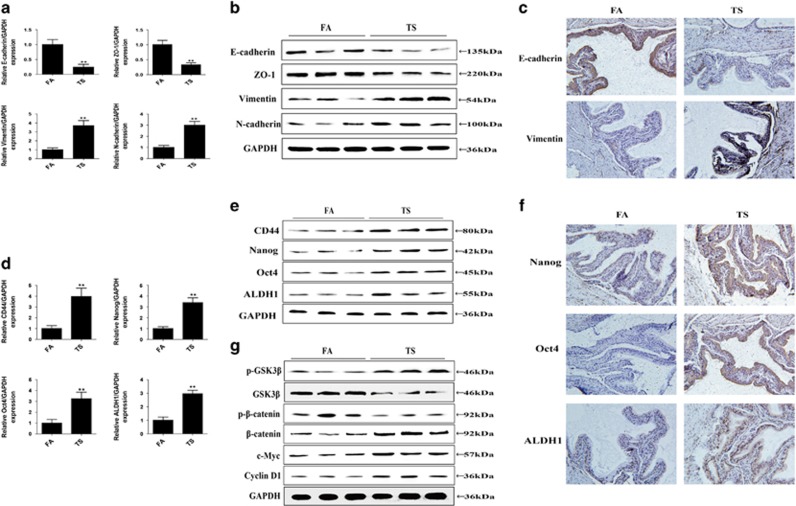
TS-induced alterations in EMT and CSC markers expression and activated Wnt/*β*-catenin in the mice bladder exposed to TS for 12 weeks. (**a**-**c**) TS decreased the levels of epithelial markers, and increased the expression of mesenchymal markers. (**d**-**f**) TS elevated the expression of specific cell surface markers of CSCs. (**g**) TS increased p-GSK3*β*, *β*-catenin, c-Myc and cyclin D1 expression levels and reduced the expression of GSK3*β* and p-*β*-catenin. **P<0.01, compared with FA

We investigated whether TS induces EMT-like changes and acquisition of CSCs properties in an animal model. BALB/c mice were exposed to TS for 12 weeks, and then the expression levels of EMT markers E-cadherin, ZO-1, Vimentin and N-cadherin in bladder tissues were examined. Results revealed that TS exposure decreased the mRNA and protein expression levels of E-cadherin and ZO-1, and elevated expression levels of Vimentin and N-cadherin in the mice bladder (Figures 6a and c). To further determine the effect of TS exposure on the CSCs characteristics of bladder cells, the expression levels of several CSCs markers including CD44, Nanog, Oct4 and ALDH1 were examined. As illustrated in Figures 6d and f, the mRNA and protein levels of bladder CSCs markers were significantly upregulated after 12 weeks TS exposure.

We also evaluate whether TS-elicited bladder EMT alterations and the CSCs characteristics are associated with changes in Wnt/*β*-catenin activation, the expression levels of p-GSK3*β*, GSK3*β*, p-*β*-catenin, *β*-catenin, c-Myc and cyclin D1 were measured. It was found that TS activated Wnt/*β*-catenin pathway (Figure 6g).

### Wnt/*β*-catenin inhibition attenuated TS-triggered EMT and CSCs features in mice bladders

**Figure 7:**
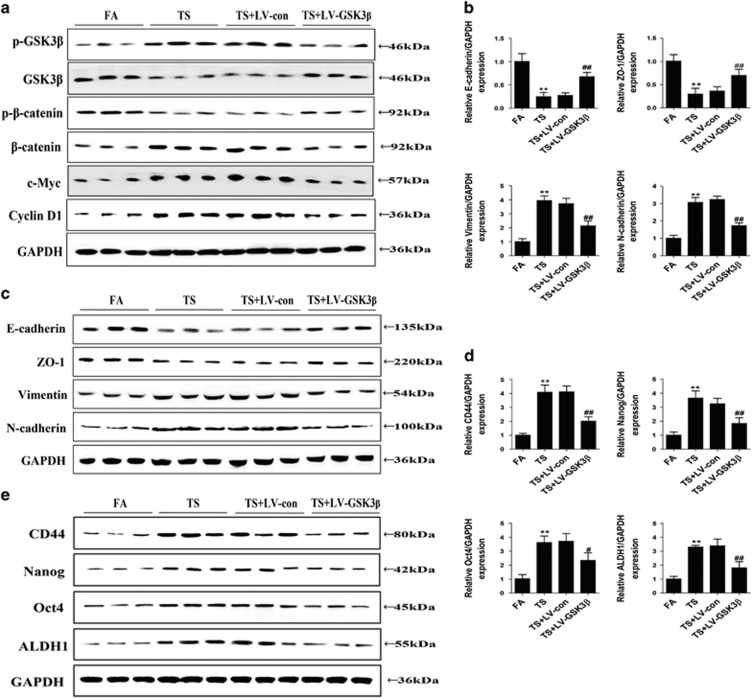
Wnt/*β*-catenin inhibition attenuated TS-triggered EMT and CSCs features after exposed to TS for 12 weeks. (**a**) Delivery of lentiviral GSK3*β* expression vector inhibited Wnt/*β*-catenin activation in the bladder tissues of mice exposed to TS for 12 weeks. (**b** and **c**) Lentivirus-mediated Wnt/*β*-catenin suppression attenuated TS reduced the levels of epithelial markers, and increased the expression of mesenchymal markers. (**d** and **e**) Wnt/*β*-catenin inhibition suppressed TS-elevated expression of CSCs markers in mouse bladders. **P<0.01, compared with control group; ^#^*P*<0.05, ^##^*P*<0.01, compared with TS groups

In order to investigate the regulation of Wnt/*β*-catenin in TS-mediated EMT and CSC-like properties *in vivo*, BALB/c mice were intratracheally delivered with GSK3*β* overexpression lentiviral vector and exposed to TS for 12 weeks. Western blot analysis results showed that TS-decreased GSK3*β* activation and TS-triggered activation of *β*-catenin, c-Myc and cyclin D1 in mice bladders was restored by the delivery of GSK3*β* vector (Figure 7a). TS-induced alterations in the expression of the EMT markers, including decrease of E-cadherin and ZO-1, and increases of Vimentin and N-cadherin, were effectively attenuated by GSK3*β* vector delivery (Figures 7b and c). Moreover, GSK3*β* overexpression decreased the protein and mRNA expression of CSCs markers including CD44, Nanog, Oct4 and ALDH1 (Figures 7d and e).

### Curcumin prevented TS-induced EMT and CSCs properties though Wnt/*β*-catenin

**Figure 8:**
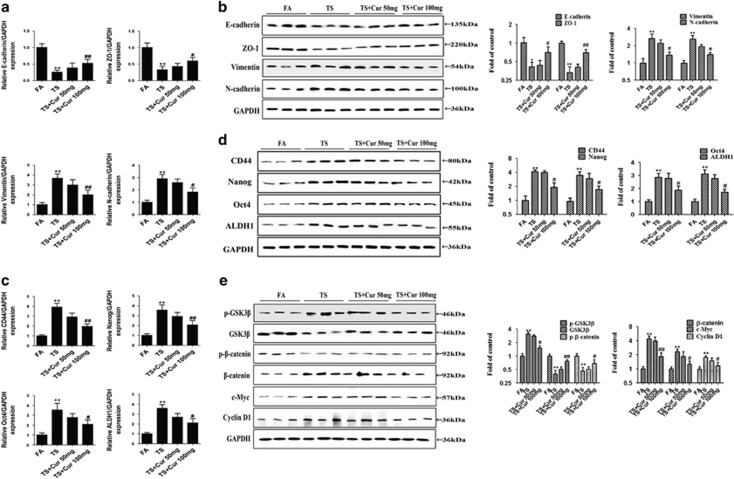
Curcumin suppressed Wnt/*β*-catenin to prevent CSE-induced urocystic EMT and stemness. (**a** and **b**) Curcumin reversed TS-induced reduce of the levels of epithelial markers, and increased the expression of mesenchymal markers. (**c** and **d**) Curcumin prevented TS-induced increases in the mRNA levels of CSCs markers. (**e**) Curcumin ameliorated TS-induced activation of Wnt/*β*-catenin. *P<0.05, **P<0.01, compared with control group; ^#^*P*<0.05, ^##^*P*<0.01, compared with TS groups

In order to determine the effects of curcumin on TS-mediated EMT and CSC-like properties in the bladder tissues, mice received curcumin (50 or 100 mg/kg body weight (BW)) and were exposed to TS. Figures 8a and b show that TS-induced alterations in the mRNA and protein levels of the EMT markers, including E-cadherin, ZO-1, Vimentin and N-cadherin, were effectively reversed by curcumin (100 mg/kg BW). Curcumin treatment also significantly decreased TS-induced expression levels of CSCs markers (CD44, Nanog, Oct4 and ALDH1). These data indicated that curcumin reversed TS-induced urocystic EMT and CSC-like *in vivo.* To explore the influence of curcumin on TS-mediated urocystic activation of Wnt /*β*-catenin pathway, we further examined the changes in Wnt/*β*-catenin activation following curcumin treatment. Western blot analyses showed that curcumin restored TS-suppressed GSK3*β* activity and suppressed TS-induced *β*-catenin and c-Myc activation in a dose-dependent manner.

## Discussion

Bladder cancer is a socially significant healthcare problem and accounting for estimated 80 500 new cases diagnosed and 32 900 deaths in China. Both environmental and genetic factors are important in bladder carcinogenesis.^29, 30^ The relationship between bladder cancer and TS has been established for TS is one of the leading causes of bladder cancer.^29, 30^ However, the exact mechanisms by which TS causes the occurrence of bladder cancer remain unknown. In this study, we revealed that long time TS exposure induced bladder cells malignant transformation, EMT alterations and acquisition of stemness properties *in vitro* and *in vivo*. We further showed that chronic exposure of TS-induced bladder EMT and acquisition of CSC-like properties were associated with activation of Wnt/*β*-catenin signaling pathway. Meanwhile, our data indicated that curcumin attenuated TS triggered Wnt/*β*-catenin pathway activation and effectively prevented TS-induced urocystic EMT and acquisition of cancer CSC-like properties. These findings provide new insights into the pathogenesis and the chemoprevention of TS-associated bladder cancer.

To investigate the mechanisms of bladder tumorigenesis induced by TS, we used CSE to mimic the effects of TS *in vitro*. The viabilities of SV-HUC-1 cells exposed to CSE at concentrations of 0, 0.05, 0.1, 0.25, 0.5, 1 or 2% were examined. As shown in Figure 1a, there were substantial decrease in the viabilities of cells exposed to CSE at a concentration of 2% for 14 days. Thus, CSE at concentrations of 0.5 and 1% were selected for repeated, long-term exposure of cells (about 40 passages). These concentrations of CSE resulted in neoplastic transformation of SV-HUC-1 cells, as determined by independent clone formation, doubling time and nude mouse xenograft models. These results suggested that CSE-induced malignant transformation of SV-HUC-1 cells.

EMT is an important process contributing to cellular transdifferentiation, the progress of cancer and the invasion/metastasis, consisting in the loss of epithelial properties and the gain of mesenchymal characteristics.^12^ Evidences have suggested that, in addition to facilitating tumor invasion and metastasis, EMT is also critically involved in the cell transformation and initiation of tumorigenesis.^28, 29, 30, 31, 32, 33, 34^ As described in this study, chronic TS exposure induced the EMT in SV-HUC-1 cells and the mice bladders, as indicated by alterations in the mRNA and protein expression levels of the EMT markers, including E-cadherin, ZO-1, Vimentin and N-cadherin. Immunofluorescent staining and immunohistochemistry also showed that long-term TS exposure decreased E-cadherin expression and increased Vimentin expression. The results suggested that TS-triggered urocystic EMT was associated with cell malignant transformation.

CSCs have an indispensable role in the formation, progress recurrence and metastasis of human tumors because of the highly tumorigenic, self-renewal and differentiation capabilities. CSC-like cells have been identified based on the expression of different cellular CSC markers. CD44, a specific receptor for hyaluronic acid and adhesion/homing molecule, is a common bladder CSCs surface marker.^35^ It is reported that CD44+ bladder cancer cells represent enhanced capability of tumorigenic potential both *in vitro* and *in vivo*.^35, 36^ ALDH1 is also confirmed to be one of the distinct bladder CSCs markers.^35^ High expression of ALDH1 was markedly associated with an advanced tumor grade, frequent tumor recurrence and poor prognosis.^37^ It has been reported that the pluripotent stem cell factors Nanog and Oct4 characterizing CSCs properties in many cancers including bladder cancer.^38, 39^ Consistent with the previous reports, our present study shows significantly increased expression levels of these bladder CSCs markers (CD44, ALDH1, Nanog and Oct4) in chronic TS exposed SV-HUC-1 cells and the mice bladder, suggesting that these cells possess some CSCs traits.

Empirical evidence concerning the connection of EMT program to CSCs has been reported recently. As it has been reported previously, cancer cells that underwent EMT acquired characteristics of CSCs. Several lines of evidence have shown that CSCs represent a plastic state of tumor cells undergoing EMT process and exhibit a mesenchymal-like appearance.^13, 40^ EMT and stemness are both extremely important characteristics for cells to acquire more invasive and metastatic potential. In this study, we also found that chronic TS exposure reinforces CSCs and EMT during the malignant transformation of bladder cells.

Recent studies have focused on the signaling pathways mediated EMT and stemness. In many cancers, Wnt/*β*-catenin pathway is constitutively active and promotes EMT and loss of Wnt/*β*-catenin pathway is associated with inhibition of CSCs stemness. In our study, we showed that Wnt/*β*-catenin pathway may have an important role in the long time TS exposure induced EMT and acquisition of CSC-like properties *in vitro* and *in vivo*. To determine the role of Wnt/*β*-catenin pathway in TS-induced EMT and acquisition of CSC-like properties, the activation effect of TS on Wnt/*β*-catenin was mimicked with LiCl, a specific Wnt/*β*-catenin pathway activator. Activation of Wnt/*β*-catenin alone resulted in EMT-like changes and alterations in CSCs markers expression. Furthermore, we illustrated that overexpression GSK3*β* by lentivirus abolished acquisition of CSC-like properties and EMT changes, indicating the essential role of Wnt/*β*-catenin pathway in these processes.

Tumor chemoprevention, that is, using natural, synthetic or biologic chemical agents to reverse, suppress or prevent the process of carcinogenesis, has been shown to be a very promising approach to prevent cancer development, especially in high-risk populations. Curcumin, a yellow coloring agent, is the principal active component of the famous spice turmeric. It has long been used for medical purposes and food additive in China and India. Curcumin is one of the most promising chemopreventive agents. Various animal and human studies have shown that curcumin is safe and excellent tolerance when administered systemically.^41, 42, 43, 44, 45, 46^ Large amount of studies from *in vitro and in vivo* have indicated that curcumin exerts antioxidant, antiinflammatory, anticancer and antifibrotic properties..^26, 47^ In recent years, the ability of curcumin to target CSCs has been reported in a number of *in vitro* studies.^24, 25^ Evidence has revealed that curcumin modulate associated pathways. In this study, we found that curcumin (100mg/kg BW) inhibited the activation of Wnt/*β*-catenin pathway in chronic TS exposed bladder cells, as evidenced by increased GSK3*β*, decreased *β*-catenin and its downstream gene c-Myc, cyclin D1. Moreover, we demonstrated that the suppressive effects of curcumin on EMT process and changes of CSC markers expression. Taken together, these data indicated the interventional effect of curcumin on chronic TS exposure-mediated urocystic EMT and acquisition of CSC-like properties via Wnt/*β*-catenin inhibition.

In summary, this study demonstrated the role of Wnt/*β*-catenin pathway in regulating long-term TS exposure-triggered EMT and acquisition of CSCs properties and the protective effects of curcumin on chronic TS exposure mediated urocystic EMT and acquisition of CSCs traits via Wnt/*β*-catenin pathway suppression. These findings may provide new insights into the molecular mechanism of TS-associated bladder tumorigenesis and the curcumin intervention.

## Materials and methods

See the Supplementary material for further details concerning methods.

### Cell culture and chronic TS exposure

T24 cells were obtained from American Type Culture Collection (ATCC, Rockville, MD, USA) and maintained in RPMI-1640 medium. SV-HUC-1 cells were obtained from the Chinese Academy of Typical Culture Collection Cell Bank (Shanghai, China). Cells were cultured in F12K medium. CSE was prepared daily according to the reported method.^48, 49^ One filterless Hongtashan cigarette, one of the most consumed cigarette in China, was combusted and used for preparation of 10 ml F12K medium, which was referred to as a 100% CSE solution. SV-HUC-1 cells were exposed to various concentrations of CSE for about 40 passages.

### Independent clone formation

To test their capacity for independent clone formation, the chronically CSE exposed SV-HUC-1 cells or control cells were plated at a density of 500 cells in 1 ml of F12K medium and medium was changed every 3 days.

### Transfection of GSK3*β* overexpression lentiviral vectors

The chronically CSE exposed SV-HUC-1 cells were stably transfected with overexpression lentiviral vector for GSK3*β* or the negative control vector according to the manufacturer’s protocol.

### Mice and exposure to TS

Male BALB/c mice (6–8 weeks, 18–22 g) were purchased from the Animal Research Center of Jiangsu University. Mice were handled in accordance with the recommendations in the guidelines of Laboratory Animal Management Committee of Jiangsu University. Mice were allowed to 1-week acclimating to circumstances and then randomly assigned into each group (*n*=6). Mice were exposed to filtered air or TS with a target concentration of total particulate matter of 85 mg/m^3^ for 6 h per day for 12 weeks. After the last TS exposure, mice were killed and bladder tissues were collected, frozen and stored at −80 °C for further experiments.

### *In vivo* delivery of GSK3*β* overexpression lentiviral vectors

In another separate animal study, mice were randomly divided into four groups (*n*=8): filtered air group; TS-exposed group, mice were exposed to TS; TS+LV-control group, mice were delivered with negative control lentiviral vector and exposed to TS; TS+LV-GSK3*β* group, mice were delivered with GSK3*β* overexpression lentiviral vector and exposed to TS. The intratracheal delivery of lentiviral vectors was performed every 4 weeks and mice were exposed to filtered air or TS for 12 weeks.

### Curcumin treatment of mice

Mice were treated with 50 or 100 mg/kg BW curcumin per day. Before feeding, curcumin was dissolved with corn oil. Animals were randomly assigned into each group (*n*=8): control group, mice were exposed to air and received control diet containing corn oil; TS group, mice were exposed to TS and received control diet containing corn oil; TS+Cur 50 mg/kg group, mice were exposed to TS and received diet supplemented with curcumin at dose of 50 mg/kg BW per day; TS+Cur 100 mg/kg group, mice were treated with 100 mg/kg BW curcumin and exposed to TS.

### Tumorigenicity in nude mice

Four-week old BALB/c nude mice were randomly divided into two groups (four mice per group). Mouse xenograft assays were performed to detect the degree of malignancy of the chronic TS exposed cells.

### Western blot analysis

After chronic exposure, proteins were extracted from SV-HUC-1 cells and mouse bladder tissues. Western blot analysis were performed for the determination of target protein expression levels.

### Quantitative reverse transcriptase-polymerase chain reaction

qRT-PCR analyses were performed using the Power SYBR Green Master Mix by an ABI 7300 real-time PCR detection system to determine the levels of target genes. The primers used were as follows: E-cadherin forward primer 5′-TCGACACCCGATTCAAAGTGG-3′ and reverse primer 5′-TTCCAGAAACGGAGGCCTGAT-3′ ZO-1 forward primer 5′-GCAGCCACAACCAATTCATAG-3′ and reverse primer 5′-GCAGACGATGTTCATAGTTTC-3′ Vimentin forward primer 5′-CCTTGACATTGAGATTGCCA-3′ and reverse primer 5′-GTATCAACCAGAGGGAGTGA-3′ N-cadherin forward primer 5′-ATCAAGTGCCATTAGCCAAG-3′ and reverse primer 5′-CTGAGCAGTGAATGTTGTCA-3′ CD44 forward primer 5′-AGCCCATGTTGTAGCAAACC-3′ and reverse primer 5′-TGAGGTACAGGCCCTCTGAT-3′ Oct4 forward primer 5′-GTGGAGAGCAACTCCGATG-3′ and reverse primer 5′-TGCTCCAGCTTCTCCTTCTC-3′ Nanog forward primer 5′-CCTCTCCGCTTCCTTCCT-3′ and reverse primer 5′-CTGTTTGTAGCTAAGGTTCAGGAGG-3′ ALDH1 forward primer 5′-TGGCTGATTTAATCGAAAGAGAT-3′ and reverse primer 5′-TCCACCATTCATTGACTCCAPMID-3′ GAPDH forward primer 5′-GCTGCCCAACGCACCGAATA-3′ and reverse primer 5′-GAGTCAACGGATTTGGTCGT-3′.

### Immunofluorescent staining

Immunofluorescent staining was performed to analyze the expression of E-cadherin, Vimentin, Nanog and OCT4 in CSE-transformed SV-HUC-1 cells.

### Immunohistochemistry

Following the completion of exposure, mice were killed and bladder tissues were collected and then the levels of E-cadherin, Vimentin, Nanog, OCT4 and ALDH1 were determined by immunohistochemistry.

### Statistical analysis

Statistical analyses were performed with SPSS 16.0 (SPSS, Inc., Chicago, IL, USA). All data were expressed as mean±S.D. One-way ANOVA was used for comparison of statistical differences among multiple groups, followed by the LSD significant difference test. In case of comparison between two groups, unpaired Student's *t*-test was used. A value of *P*<0.05 was considered significantly different.
