# Supplementary material for: Curcumin reversed chronic tobacco smoke exposure induced urocystic EMT and acquisition of cancer stem cells properties via Wnt/β-catenin
Source: Cell Death Dis. 2017 Oct 5;8(10):e3066–. doi: 10.1038/cddis.2017.452 (PMC5680574; doi:10.1038/cddis.2017.452)
Supplement: Supplementary Information [file cddis2017452x1.docx]

**Online Supplement**

**Materials and methods**

**Chemicals and Reagents**

E-cadherin, N-cadherin, GSK3β, β-catenin, c-Myc, N-cadherin, Vimentin, CD44, Nanog, and ALDH1 antibodies were from Cell Signaling Technology (Beverly, MA). Histone and ZO-1 were from Santa Cruz Biotechnology (Santa Cruz, CA). p-GSK3β, p-β-catenin, Cyclin D1, Oct4 were from Proteintech Group (Chicago, IL). GAPDH was from Biogot Technology (Nanjing, China). E-cadherin, ZO-1, N-cadherin, Vimentin, CD44, Nanog, Oct4, ALDH1 and GAPDH primers were synthesized according to published sequences from Invitrogen (Carlsbad, CA). Cell culture reagents were purchased from Gibco (Carlsbad, CA). Curcumin was purchased from Sigma-Aldrich (St Louis, MO, USA, purity > 98.0%). Sources of other materials are mentioned in correspondingly in the text.

**Cell culture**

The SV-40 immortalized human urothelial cell line (SV-HUC-1) was obtained from the Chinese Academy of Typical Culture Collection Cell Bank (Shanghai, China). Cells were cultured in F12K medium. Cell cultures were maintained in a humidified incubator at 37°C with 5% CO_2_. Medium was changed every other day until SV-HUC-1 cells reached about 80% confluence. The experiments were performed with SV-HUC-1 cells between passages 5-7.

**Preparation of cigarette smoke extract**

Cigarette smoke extract (CSE) was prepared daily according to the reported method. Briefly, one filterless Hongtashan cigarette, one of the most consumed cigarette in China (12 mg tar and 1.1mg nicotine per cigarette) was smoked and the smoke was continuously drawn through a glass syringe containing 10 ml of FBS-free F12K medium at a rate of 5min/per cigarette to generate CSE solution. The CSE suspension was adjusted to pH 7.4 and filtered through a 0.22-μm-pore size filter. The obtained CSE solution was referred to as 100% and then diluted to the desired concentrations with F12K medium.

**Chronic TS exposure**

In our previous studies, we found that cell viability was not apparently affected in cells treated with CSE at concentrations up to 2% for 14 days. Therefore, 1% CSE was selected as the maximum concentration for the long time exposure. For chronic exposure, 1×10^6^ SV-HUC-1 cells were seeded into dishes for 12-24 h, and then exposed to CSE (0%, 0.5%, 1%) for about 48-72 h per passage. The medium containing various concentrations of CSE was changed every day. This process was continued for about 40 passages.

**Independent clone formation**

To test their capacity for independent clone formation, the chronically CSE exposed SV-HUC-1 cells or control cells were plated at a density of 500 cells in 1 ml of F12K medium and medium was changed every three days. After 14 days, the colonies were observed under a microscope.

**Transfection of GSK3β overexpression lentiviral vectors**

The chronically CSE exposed SV-HUC-1 cells were stably transfected with overexpression lentiviral vector for GSK3β or the negative control vector according to manufacture’s protocol. Briefly, SV-HUC-1 cells were cultured in F12K medium and infected with lentiviral vector at multiplicity of infection of 5, 10, 15, 30 and 50. The infection medium was removed and replaced by fresh medium after overnight incubation. The transfection efﬁcacy was determined 72 h later by Green fluorescent protein fluorescence imaging using ﬂuorescent microscopy. Transfection assays were accomplished at a multiplicity of infection of 30, the optimal infection efficiency.

**Mice and exposure to TS**

Male BALB/c mice (6-8 weeks old, 18-22g) were purchased from the Animal Research Center of Jiangsu University. Mice were housed in standard clear plastic cages with free access to water and basal diet, maintained at 22±2°C and 40-60% relative humidity with a 12/12hours light/dark cycle. Mice were handled in accordance with the recommendations in the guidelines of the Animal Care and Welfare Committee of Jiangsu University.

Mice were allowed to one week acclimating to circumstances and then randomly assigned into each group (n=6). Mice in the control group were exposed to filtered air. Mice in the TS exposed group were exposed for 6 hours per day for 12 weeks. Hongtashan (contains 12 mg tar and 1.1mg nicotine/cigarette), one of the most consumed cigarette in China, was used as research cigarettes in our present study. TS was generated by a smoke machine which smoked the filterless cigarettes at a constant rate (5min per cigarette) and then delivered the mainstream smoke to the whole-body exposure chambers with target concentration of total particulate matter (TPM) of 85 mg/m^3^. The exposure conditions for each group were monitored and characterized as the followings: carbon monoxide (14.92±2.25 ppm), TPM (0 mg/m^3^) for the control group; carbon monoxide (179.43±10.88 ppm), TPM (83.93±4.38 mg/m^3^) for TS exposure group. After the last TS exposure, mice were sacrificed and bladder tissues were collected, frozen and stored at -80°C for further experiments.

***In vivo* delivery of GSK3β overexpression lentiviral vectors**

In a separate set of animal study, mice were randomly divided into four groups (n=8 per group): filtered air group; TS-exposed group, mice were exposed to TS; TS+LV-control group, mice were delivered with negative control lentiviral vector and exposed to TS; TS+LV-GSK3β group, mice were delivered with GSK3β overexpression lentiviral vector and exposed to TS. In the lentivirus groups, mice were delivered with negative control lentiviral vector or GSK3β overexpression lentiviral vectors. In brief, mice were anesthesized using intraperitoneal injections of 10% chloral hydrate (3 µl/ g body weight). After disinfection, a vertical incision (<1.0 cm) was made in the mice abdomen and the bladder was exposed. Negative control lentiviral vector or GSK3β lentiviral vectors were injected into the bladder muscle, final 10-20 points on the muscle were injected with 2×10^7^ vectors. Upon completion of the instillation, the skin and fascia were closed in one layer with interrupted sutures. The intratracheal delivery of lentiviral vectors was performed every four weeks and mice were exposed to filtered air or TS for 12 weeks. After the complection of exposure, mice were sacrificed and bladder tissues were collected for analysis.

**Curcumin treatment of mice**

Mice were treated with 50 or 100 mg/kg body weight (BW) curcumin per day. Prior to feeding, curcumin was dissolved with corn oil. Animals were randomly divided into four groups (n=8 per group): filtered air group, mice were exposed to filtered air and received control diet containing corn oil; TS-exposed group, mice were exposed to TS and received control diet containing corn oil; TS+curcumin 50 mg/kg group, mice were exposed to TS and received control diet supplemented with curcumin at dose of 50 mg/kg BW per day; tobacco smoke+curcumin 100 mg/kg group, mice were treated with 100 mg/kg BW per day curcumin and exposed to TS. The administration dosages of curcumin were based on the measurements of mouse body weight and the amount of diet consumption. After 12 weeks exposure, mice were sacrificed, and the bladder tissues were collected and stored for further experiments.

**Tumorigenicity in nude mice**

Four-week old BALB/c nude mice (Laboratory Animal Center of Shanghai, Academy of Science, Shanghai, China) were randomly divided into 2 groups (4 mice /group). Mouse xenograft assays were performed to detect the degree of malignancy of the chronic TS exposed cells. Briefly, 1 × 10^7^ chronic TS treated SV-HUC-1 cells were injected subcutaneously into the front portion of two sides of mice. There were 6 mice per group. Tumor incidence was monitored once per week.

**Western blot analysis**

After chronic exposure, SV-HUC-1 Cells were washed twice with ice-cold PBS and cell proteins were extracted in a lysate buffer and then centrifuged at 4°C for 20 min. In animal studies, bladder tissues were homogenized in a lysate buffer and then centrifugation at 12,000 rpm for 20 min to obtain the final supernatants. Equal amounts of proteins were fractionated by separated by gel electrophoresis using a 7.5 or 10% SDS-PAGE, depending on the size of the protein being detected and then transferred to PVDF membrane (Millipore, Billerica, MA). The membranes were blocked with 5% defatted milk for 1 hour at room temperature with mild agitation and subsequently incubated with monoclonal antibody overnight at 4°C. The membranes were washed 3 x 10 minutes with Tris-buffered saline Tween and then probed with horseradish peroxidase-conjugated secondary antibody. GAPDH served as the loading control.

**Quantitative reverse transcriptase-polymerase chain reaction**

In cell and animal studies, total RNA was isolated from cells and frozen bladder tissues using the RNAiso Plus according to the manufacturer’s instructions (TaKaRa, Japan). The purity of the extracted RNA was measured by UV spectrophotometry (260/280 nm ratio). Two micrograms RNA was reverse transcribed into cDNA using AMV Reverse Transcriptase (Promega, Madison, WI). qRT-PCR was performed using the Power SYBR Green Master Mix (TaKaRa, Japan) by an ABI 7300 real-time PCR detection system (Applied Biosystems, CA). The primers used were as follows: E-cadherin forward primer 5'-TCGACACCCGATTCAAAGTGG-3' and reverse primer 5'-TTCCAGAAACGGAGGCCTGAT-3'; ZO-1 forward primer 5'-GCAGCCACAACCAATTCATAG-3' and reverse primer 5'-GCAGACGATGTTCATAGTTTC-3'; Vimentin forward primer 5'-CCTTGACATTGAGATTGCCA-3' and reverse primer 5'-GTATCAACCAGAGGGAGTGA-3'; N-cadherin forward primer 5'-ATCAAGTGCCATTAGCCAAG-3' and reverse primer 5'-CTGAGCAGTGAATGTTGTCA-3'; CD44 forward primer 5'AGCCCATGTTGTAGCAAACC3' and reverse primer 5'TGAGGTACAGGCCCTCTGAT3'; Oct4 forward primer 5'GTGGAGAGCAACTCCGATG3' and reverse primer 5'TGCTCCAGCTTCTCCTTCTC3'; Nanog forward primer 5'CCTCTCCGCTTCCTTCCT3' and reverse primer 5'CTGTTTGTAGCTAAGGTTCAGGAGG3'; ALDH1 forward primer 5' -TGGCTGATTTAATCGAAAGAGAT3' and reverse primer 5' -TCCACCATTCATTGACTCCAPMID3'; GAPDH forward primer 5'-GCTGCCCAACGCACCGAATA-3' and reverse primer 5'-GAGTCAACGGATTTGGTCGT-3'. The mRNA expression level for each gene was normalized by its respective GAPDH. Fold changes in gene expression were calculated by a comparative threshold cycle (Ct) method using the formula 2^− (ΔΔC t)^.

**Immunofluorescent staining**

The long term CSE exposed SV-HUC-1 cells were immobilized and stained with rabbit E-cadherin, Vimentin, Nanog and OCT4 antibodies at 4°C overnight. After washing with Tris-buffered saline Tween, SV-HUC-1 cells were incubated with FITC-conjugated secondary antibody (Leinco Technology, St Louis, MO) at room temperature. 4′, 6-diamidino-2-phenylindole (DAPI, Sigma, St. Louis, MO) was added for 10-15 min to stain the nuclei. After the completion of staining, images were captured via a fluorescence microscope. (Zeiss, LSM700B, Germany).

**Immunohistochemistry**

Following the completion of exposure, mice were sacrificed and bladder tissues were collected and then immunohistochemistry was performed. Briefly, 5μm serial paraffin-embedded bladder sections were de-waxed in xylene and rehydrated in graded alcohol, after which endogenous peroxidase activity was quenched with exposure to 3% (v/v) H_2_O_2_ diluted with methanol for 10min. The sections were boiled in citrate buffer (pH 6.0, 10mM) to antigen retrieval. Then the sections were blocked with 5% (w/v) BSA to block the non-specific binding. After overnight incubation with the primary antibody (E-cadherin, Vimentin, Nanog, OCT4 and ALDH1) at 4°C, the sections were subsequently washed with PBS before incubation with biotinylated goat anti-rabbit immunoglobulin G for 30 min. Finally, sections were visualized with 3,3′-diaminobenzidine and then counterstained with hematoxylin for examination by light microscopy (400×).

**Statistical analysis**

Statistical analyses were performed with SPSS 16.0. All data were expressed as mean ± standard deviation. One-way ANOVA was used for comparison of statistical differences among multiple groups, followed by the LSD significant difference test. In case of comparison between two groups, unpaired Student t test was used. A value of p < 0.05 was considered significantly different.
